# Archaeal Orc1 protein interacts with T-rich single-stranded DNA

**DOI:** 10.1186/s13104-021-05690-w

**Published:** 2021-07-19

**Authors:** Katarzyna Wegrzyn, Igor Konieczny

**Affiliations:** Intercollegiate Faculty of Biotechnology of University of Gdansk and Medical University of Gdansk, University of Gdansk, Abrahama 58, 80-307 Gdansk, Poland

**Keywords:** DNA replication initiator, Protein-DNA interaction, *Aeropyrum pernix*, DNA unwinding element

## Abstract

**Objective:**

The ability to form nucleoprotein complexes is a fundamental activity of DNA replication initiation proteins. They bind within or nearby the region of replication origin what results in melting of a double-stranded DNA (dsDNA) and formation of single-stranded DNA (ssDNA) region where the replication machinery can assemble. For prokaryotic initiators it was shown that they interact with the formed ssDNA and that this interaction is required for the replication activity. The ability to interact with ssDNA was also shown for *Saccharomyces cerevisiae* replication initiation protein complex ORC. For Archaea, which combine features of both prokaryotic and eukaryotic organisms, there was no evidence whether DNA replication initiators can interact with ssDNA. We address this issue in this study.

**Results:**

Using purified Orc1 protein from *Aeropyrum pernix* (*Ap*Orc1) we analyzed its ability to interact with ssDNA containing sequence of an AT-rich region of the *A. pernix* origin *Ori1* as well as with homopolymers of thymidine (polyT) and adenosine (polyA). The Bio-layer interferometry, surface plasmon resonance and microscale thermophoresis showed that the *Ap*Orc1 can interact with ssDNA and it binds preferentially to T-rich ssDNA. The hydrolysis of ATP is not required for this interaction.

**Supplementary Information:**

The online version contains supplementary material available at 10.1186/s13104-021-05690-w.

## Introduction

The key steps of replication of genetic material are similar for both prokaryotes and eukaryotes, however, there are important differences distinguishing these processes in different kingdoms of life. In prokaryotes there are well defined regions of replication origin, where specific motifs are recognized initially by the replication initiation proteins. Binding of initiators results in melting within the AT-rich DNA unwinding element (DUE) [1–3]. Bacterial and plasmid replication initiators, despite binding the double-stranded DNA (dsDNA), also interact with the single-stranded DNA (ssDNA) produced in DUE [4–6]. For bacterial DnaA replication initiator this interaction takes place via the AAA+ (ATPases associated with various cellular activities) domain and depends on protein bound with ATP [7]. In eukaryotic cells these early steps of DNA replication are more complex [8, 9]. Except for *Saccharomycotina* species, in which the replication origins contain distinguishable sequence motifs, the eukaryotes’ origins do not contain consensus DNA sequence elements [10]. Proteins of human Origin Recognition Complex (ORC) bind in vitro both origin and non-origin sequences equally well [11]. Although it was shown for human and *Drosophila* ORC that they bind preferentially to AT-rich dsDNA [11–13], the initiation of DNA replication seems to be determined rather by DNA topology, structural features and the chromatin environment [13, 14]. Nevertheless, the formation of pre-replication complex (pre-RC), containing helicase complex (MCM, minichromosome maintenance proteins) loaded onto DNA molecule, requires, as in prokaryotes, replication initiator proteins—ORC [15]. There is no evidence for interaction of the metazoan ORC with ssDNA within the melted region of origin. However, the electrophoretic mobility shift assay (EMSA) with the use of purified entire *Saccharomyces cerevisiae* ORC protein complex and ssDNA fragment containing sequence of *ARS1* showed that the ORC binds ssDNA, but only in a presence of Orc1p protein [16]. It was proposed that the interaction with ssDNA can occur during replication initiation process [16].

In Archaea, the DNA replication initiation may vary in different species, due to diverse replication machineries and diverse structures of origins [17], (Additional file 1: Figure S1). It also combines some features of both bacterial and eukaryotic replication factories. Among Archaea, species with one (like in bacteria) or numerous (as in eukaryotes) replication origins can be found [18]. Although the archaeal replication initiators are homologues of the eukaryotic Orc1 and Cdc6 proteins from ORC, they bind specific motifs (ORB, origin recognition box) within the origin, as the bacterial initiators do. In species containing just one replication origin e.g. *Pyroccocus furiosus*, only one Orc1 protein is encoded in the proximity of the origin. Species possessing more than one origin e.g. *Sulfolobus solfataricus*, encode multiple Orc1 paralogues [19].

**Fig. 1:**
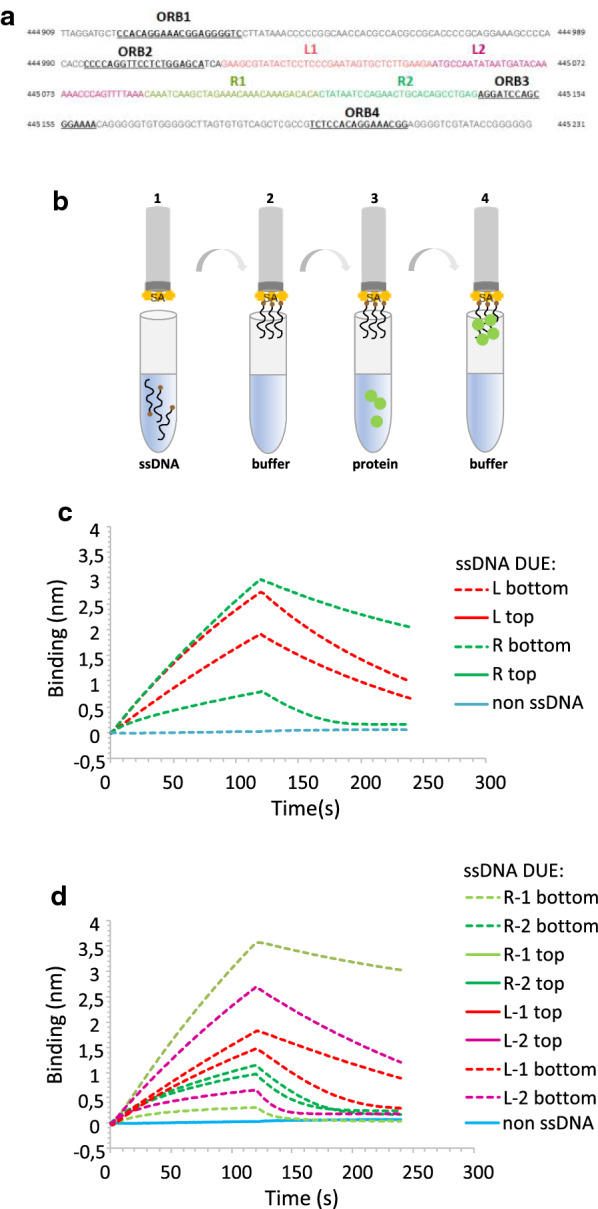
*Ap*Orc1 protein binds preferentially bottom strand of the AT-rich region of *Ori1*. **A** DNA sequence of the top strand of *A. pernix Ori1* region (chromosome sequence coordinates: 444,909–445,231). ApOrc1 binding sites (Origin Recognition Box, ORB) are marked as black underlined letters. The AT-rich region is located between the ORB2 and ORB3 binding sites and is divided into four fragments: left 1 (L1) marked in red, left 2 (L2) marked in magenta, right 1 (R1) marked in light green and right 2 (R2) marked in dark green. **B** The scheme of experiment with the use of bio-layer interferometry technique. In the analysis the BLItz system with the SA biosensor was used. Biotinylated ssDNA fragments (Additional file 1: Table S1) containing sequence of the AT-rich region of *Ori1* were immobilized on biosensor surface and then incubated with the *Ap*Orc1 protein (250 nM), followed by incubation with a EDBS buffer. **C**, **D** BLItz sensograms showing binding of the *Ap*Orc1 protein to the indicated ssDNA fragments (**B**, step 3), followed by the protein’s dissociation (**B**, step 4). As a negative control, binding of *Ap*Orc1 to the SA biosensor without ssDNA was analyzed (blue curve)

*Aeropyrum pernix* chromosome contains sequence of two origin regions and encodes two Orc1 paralogues. However, only one of them (named Orc1-1 [20]) is capable of binding to the four canonical ORB binding sites [21]. This interaction results in sensitivity of the AT-rich region, located between the second and third ORB sites (Fig. 1), to digestion by P1 nuclease, suggesting that this is the sequence where melting of dsDNA occurs [21]. To date, there was no data showing if the *A. pernix* Orc1-1 or any other archaeal replication initiator can bind ssDNA, especially within the AT-rich region of origin.

Here we show the first evidence indicating the ability of *A. pernix* Orc1-1 (*Ap*Orc1) protein to interact with ssDNA, especially with T-rich sequences.

## Main text

### Materials and methods

Oligonucleotides used in this work are listed in Additional file 1: Table S1.

The details of all used methods are given in Additional file 1.

## Results and discussion

### ApOrc1 binds ssDNA within the AT-rich region of origin

To answer the question if *Ap*Orc1 protein can interact with ssDNA and if this interaction is sequence specific, first we cloned and purified His_6_-taged *A. pernix* initiator using affinity chromatography (Additional file 1: Figure S2). The basic activity of the *Ap*Orc1 protein: ability to interact with dsDNA containing specific ORB sequences (Fig. 1A) was confirmed for the purified protein using EMSA and surface plasmon resonance (SPR) (Additional file 1: Figure S3A and C). In both techniques the dsDNA fragments containing four ORBs was used. Both EMSA and SPR indicated the ability of the purified *Ap*Orc1 protein to interact with the *Ori1* sequence. In EMSA we observed shifted bands when the protein in an increasing concentrations was incubated with fluorescently labeled dsDNA. Each shifted band corresponds to a specific nucleoprotein complex and we could observe seven types of complexes Additional file 1: Figure S3A). Since it was proposed that each ORB in *Ori1* is bound by two monomers [21], in the applied experimental conditions we could observe almost full coverage of the ORBs with *Ap*Orc1. In a control experiment with non-specific dsDNA fragment we could not observe well defined retarded bands (Additional file 1: Figure S3B). In the SPR analysis we also observed an increase in the response signal when increasing concentration of *Ap*Orc1 was applied over the sensor chip surface with immobilized biotinylated dsDNA containing *Ori1* sequence (Additional file 1: Figure S3C). Next, with the use of the Bio-layer interferometry (BLI), we investigated the interaction of *Ap*Orc1 with ssDNA containing top or bottom strand sequences of the AT-rich region of *Ori1* (Fig. 1B–C). This region was previously suggested to be unwound after *Ap*Orc1-ORB sites interaction [21] and could correspond to bacterial DUE sequence bound by replication initiators. Since this region is quite long, to avoid formation of secondary structures, we separately investigated its two fragments: left (L) and right (R) (Fig. 1A). In the assay biotinylated ssDNA containing sequence of top or bottom strand of L or R fragments were immobilized on the surface of biosensors and the interaction with the *Ap*Orc1 protein was measured in real time. The highest increase in signal was detected when binding with bottom strand of the R fragment was investigated (Fig. 1C, green dashed line). The signal was much weaker for ssDNA containing sequence of top strand of R fragment (Fig. 1C, green solid line). Also for the L fragment, higher signal was observed for bottom strand (Fig. 1C, red dashed line) comparting to the top strand (Fig. 1C, red solid line). However the difference was not as significant as in case of strands of R fragment.

**Fig. 2:**
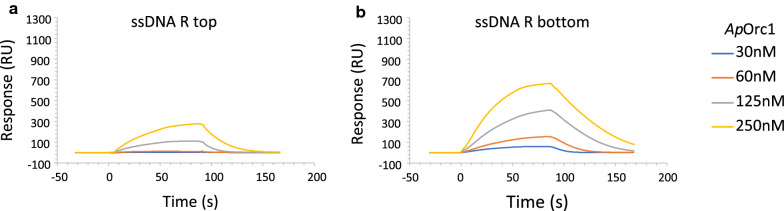
*Ap*Orc1 binds with higher affinity to the bottom strand of the R fragment. Binding of the *Ap*Orc1 protein to ssDNA fragments (Additional file 1: Table S1) containing right top (**A**) and bottom (**B**) strands’ sequence of the *Ori1* AT-rich region was analyzed with SPR technique. Biotinylated ssDNA fragments were immobilized on a surface of SA sensor chip. Injections containing the indicated concentrations of protein in HBS-EP buffer supplemented with ATP were performed. HBS-EP was used as a running buffer

Since in BLI the highest signal was detected for R bottom strand, we analyzed the interaction with the R region also with the use of SPR. This experiment also showed that the *Ap*Orc1 binds with higher affinity to the bottom strand of the R fragment (Fig. 2A–B). The response recorded for ssDNA of the R bottom strand was 3 times higher than for the R top strand. To confirm that the difference is a result of different *Ap*Orc1 affinity to top and bottom strand and not an unequal level of ssDNA immobilization on a sensor chip surface, we verified the interaction of the *Escherichia coli* ssDNA binding protein (SSB) with these ssDNA fragments. Since SSB binding to ssDNA is independent of DNA sequence [22], as expected, we did not observe significant differences in interaction between SSB and immobilized ssDNA (Additional file 1: Figure S4). This result indicated the comparable level of ssDNA immobilization for both R strands and that different level of response for *Ap*Orc1 results from different affinity of this protein to distinct ssDNA sequences.

To be more specific in determining the sequence binding preferences of the *Ap*Orc1, we divided the R and L fragments of *A. pernix Ori1* into shorter ones (R1, R2, L1 and L2) (Fig. 1A) and again applied the BLI assay. *Ap*Orc1 showed the highest affinity to the R1 bottom and L2 bottom sequences (Fig. 1D). Also the SPR analysis indicated the highest response when the protein was tested for interaction with the R1 bottom strand sequence (Additional file 1: Figure S5). For R1 top, R2 top and R2 bottom strand sequences, although same differences were observed for both BLI and SPR results, the signal obtained for all these ssDNA fragments was significantly weaker in comparison to that obtained for R1 bottom sequence. Both R1 and L2 fragments are in the middle of the AT-rich region of the *A. pernix Ori1* sequence. It is possible that at first *Ap*Orc1 binding to the dsDNA in ORB, causes melting of the origin in the AT-rich region and then the protein interacts with ssDNA in the middle of this unwound region. It might be a requirement for proper helicase loading, however, this hypothesis requires further investigation.

In bacteria binding of ssDNA is supported by the AAA+ domain of the DnaA protein and is ATP-dependent. Only the DnaA-ATP version of protein interacts with one strand of melted AT-rich region of origin [7]. In contrast, the *S. cerevisiae* ORC complex showed the ATP-independent interaction with ssDNA [16]. To investigate if *Ap*Orc1 can bind to ssDNA in an ATP-dependent manner, using the SPR, we compared its binding to the bottom R strand in the presence (Additional file 1: Figure S6A) or absence (Additional file 1: Figure S6B) of ATP. When this nucleotide was omitted from the buffer we observed significant decrease in the *Ap*Orc1 protein interaction with ssDNA. To verify if the hydrolysis of the ATP is required for *Ap*Orc1 binding to ssDNA, we also performed SPR analysis in the presence of AMP-PMP, non-hydrolysable analogue of ATP, and ADP. For both these nucleotides the obtained response was similar as for interaction in the presence of ATP (Additional file 1: Figure S6C–D). That result indicates the *Ap*Orc1 protein binding to ssDNA within the AT-rich region of *Ori1* in the presence of a nucleotide, however ATP hydrolysis is not required for this interaction. It is possible that as it is observed for bacterial initiator proteins, also in case of the archaeal Orc1 proteins, or at least in case of *A. pernix*, the AAA+ domain is involved in this interaction with ssDNA and in the presence of the nucleotide it takes on the proper conformation. However, this requires further analysis with purified domains of this protein.

### ApOrc1 protein interacts with T-rich ssDNA sequence

**Fig. 3:**
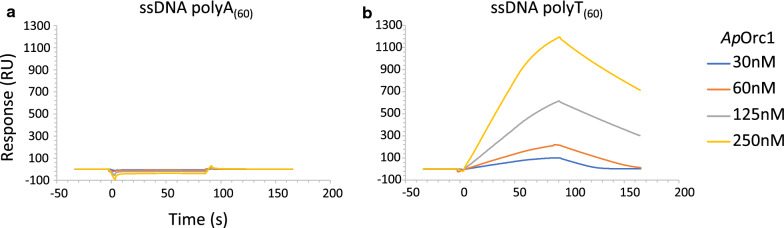
*Ap*Orc1 protein binds to T-rich ssDNA fragments. Binding of the *Ap*Orc1 protein to ssDNA fragments (Additional file 1: Table S1): homopolymers of adenosine (polyA_(60)_) (**A**) and homopolymers of thymidine (polyT_(60)_) (**B**), was analyzed with SPR technique. Biotinylated ssDNA fragments were immobilized on a surface of SA sensor chip. Injections containing the indicated concentrations of protein in HBS-EP buffer were performed. HBS-EP was used as a running buffer

Since SPR and BIL analysis indicated some preferences in *Ap*Orc1 binding to ssDNA, to further verify if the *Ap*Orc1-ssDNA interaction is sequence specific, we used ssDNA fragment containing sequence of AT-rich region of plasmid RK2 origin, that is bound by plasmid initiator [6] (Additional file 1: Figure S7). The obtained response was similar to that observed when top R sequence of *A. pernix Ori1* was used (Fig. 2A), what suggests that *Ap*Orc1 can interact to some extent with any ssDNA, however, it binds preferentially to bottom strand of the AT-rich region of the *A. pernix Ori1*. Some sequence specificity in the interaction with ssDNA was previously shown for *S. cerevisiae* ORC proteins complex [16]. Also, for other DNA replication initiators: bacterial [4, 5, 23], plasmid [6] and viral [24], it was shown that they bind to ssDNA in a sequence-specific manner. Usually the T-rich strand is bound by replication initiator as it is observed for *Ap*Orc1 (Fig. 1A). Since the R bottom strand of *Ori1* is T-rich, to test if *Ap*Orc1 binds preferentially to ssDNA with high thymidine content, we analyzed the interaction of *Ap*Orc1 with homopolymers of thymidine (polyT) and, as a control, homopolymers of adenosine (polyA), with usage of SPR (Fig. 3) and microscale thermophoresis (MST) (Additional file 1: Figure S8). In both techniques *Ap*Orc1 protein interacted with ssDNA containing thymidine (Fig. 3A, Additional file 1: Figure S8A) and there was no detected interaction when polyA was used (Fig. 3B, Additional file 1: Figure S8A). This difference was even more significant than for top and bottom R strands of *Ori1* region (Fig. 2; Additional file 1: Figure S8B), indicating the dependence of *Ap*Orc1-ssDNA complex formation on the thymidine content. The formation of *Ap*Orc1-poly(T) complex was also indicated by faint retarded band in EMSA assay (Additional file 1: Figure S9).

## Limitations

The interaction between *Ap*Orc1 and ssDNA was detected with sensitive methods as BLI, SPR and MST. However, it was challenging to detect the ApOrc1-ssDNA complex in EMSA assay, in all tested experimental conditions. The only faint retarded band was observed for *Ap*Orc1-polyT interaction (Additional file 1: Figure S9). The lack of visible complexes in EMSA, even after incubation of the samples at physiological conditions, might result from transient interaction. It cannot be excluded that within a cell there are specific factors that stabilize this complex.

## Supplementary Information

**Additional file 1: Figure S1.** Sequence alignment of archaeal origin regions. The sequences of origin regions of *A. pernix* (NC_000854), *Pyrococcus abyssi* (NC_000868), *Pyrococcus furiosus* (NC_003413) and *Sulfolobus solfataricus* (NC_003106) were chosen based on the work of Robinson and co-authors [26]. The sequence alignment was done with the use of Clustal Omega tool [27]. The sequences of the Orc proteins’ binding sites (ORB sites) are bolded. **Figure S2.** Purification of *Ap*Orc1 protein. The *Ap*Orc1 protein was purified as a His6-tagged variant from the *E. coli* Rosetta LysS strain. After overproduction of *Ap*Orc1 protein and cells lysis, a fraction of soluble proteins was applied onto a Ni–NTA resin. After the washing step, the protein bound to the resin was eluted with buffer containing 250 mM EDTA (see Materials and Methods). 5 µl of collected fractions were mixed with Laemmli buffer and separated in 12.5% polyacrylamide gel stained with Coomassie Blue dye. M—PageRuler™ Prestained Protein Ladder (Thermo Scientific) (lane 1); S—fraction of soluble proteins (lane 2); FT- fraction of proteins that did not bind to the resin (line 3); E- fractions collected during elution of proteins from the resin (lanes 4–8). **Figure S3.** Binding of *Ori1* region by *Ap*Orc1 protein. Analysis of *Ap*Orc1 protein binding to dsDNA with EMSA technique. Binding to dsDNA fragments containing sequence of Ori1 region (A) or non-specific sequence (fragment of pUC18 plasmid) (B) was tested. (A, B) Fluorescently labeled dsDNA fragment (1 pmol) was incubated with increasing concentration of *Ap*Orc1 protein (100, 200, 300 nM) and then separated in 5% PAGE in TBE buffer. Black arrows indicate the nucleoprotein complexes (A). Black asterisk indicates fuzzy band of unspecific nucleoprotein complex (B). **Figure S4.** Binding of *E. coli* SSB protein to ssDNA fragments containing sequence of top and bottom strand of the right part of the AT-rich region of *Ori1*. Binding was analyzed with SPR technique. Biotinylated ssDNA fragments (Additional file 1: Table S1) were immobilized on a surface of sensor chip SA. Injections containing the indicated concentrations of SSB protein in HBS-EP buffer were performed. HBS-EP was also used as a running buffer. **Figure S5.** Binding of *Ap*Orc1 protein to ssDNA fragments containing sequence of top and bottom strand of right part of the AT-rich region of *Ori1*. Binding of the *Ap*Orc1 protein to indicated ssDNA fragments containing sequence of right part of the AT-rich region of *Ori1* was analyzed with SPR technique. Biotinylated ssdna fragments (Additional file 1: Table S1) were immobilized on a surface of sensor chip SA. Injections containing the indicated concentrations of protein in HBS-EP buffer were performed. HBS-EP was also used as a running buffer. **Figure S6.** Influence of nucleotides on *Ap*Orc1 protein binding to ssDNA. Binding of the *Ap*Orc1 protein to ssDNA fragments (Additional file 1: Table S1) containing right bottom strands’ sequence of the Ori1 AT-rich region was analyzed with SPR technique. Biotinylated ssDNA fragments were immobilized on a surface of SA sensor chip. Injections containing the indicated concentrations of protein in HBS-EP buffer with (A) or without (B) ATP, with AMP-PMP (non-hydrolyzable analogue of ATP) (C) and ADP (D) were performed. HBS-EP was used as a running buffer. **Figure S7.** Binding of *Ap*Orc1 protein to non-specific ssDNA fragment. Binding of *Ap*Orc1 protein to ssDNA fragments (Additional file 1: Table S1) containing sequence of AT-rich region of plasmid RK2 was analyzed with SPR technique. Biotinylated ssDNA fragments were immobilized on a surface of sensor chip SA. Injections containing the indicated concentrations of protein in HBS-EP buffer were performed. HBS-EP was also used as a running buffer. **Figure S8.** MST analysis of *Ap*Orc1 binding to ssDNA fragments. Binding was analyzed with MST technique. Fluorescently labeled ssDNA fragments containing sequence of polyT(70) (A, blue) and poly A(70) (A, red) or sequence of R bottom (B, blue) and R top strands (B, red) of AT-rich region of *Ori1* (Table S1) were mixed with increasing concentration of ApOrc1 protein in EDBS buffer. Mixtures were transferred to Standard Monolith NT™ Capillaries and fluorescence was measured with Monolith NT.115. For each ssDNA fragment, at least three independent experiments were performed. The obtained data were analyzed with MO. Affinity Analysis software and presented as dependence of change in the fluorescence from protein concentration. **Figure S9.** Binding of polyT ssDNA by ApOrc1 protein. Binding of *Ap*Orc1 protein to homopolymer of thymidine (polyT_(70)_) was analyzed with EMSA technique. Fluorescently labeled ssDNA fragment (1 pmol) was incubated with increasing concentration of *Ap*Orc1 protein (100, 200, 300 nM) and then separated in 5% PAGE in TBE buffer. Black arrow indicates the nucleoprotein complex. Black asterisks indicates position of dyes from DNA loading buffer: xylene cyanol (*), bromophenol blue (**). **Figure S10.** Dependence of *Ap*Orc1 binding to R bottom ssDNA fragments from ATP concentration. Binding was analyzed with SPR technique. Biotinylated ssDNA fragments containing sequence of R bottom strand of AT-rich region of Ori1 (Additional file 1: Table S1) were immobilized on a surface of sensor chip SA. Injections containing the constant concentrations of ApOrc1 (125 nM) protein in HBS-EP buffer supplemented with increasing concentration of ATP were performed. HBS-EP was also used as a running buffer. **Table S1.** Oligonucleotides used in this study.

## Data Availability

The datasets used and/or analyzed during the current study are available from the corresponding author on reasonable request.

## Abbreviations

dsDNA: Double-stranded DNA
ssDNA: Single-stranded DNA
DUE: DNA unwinding element
polyT: Homopolymers of thymidine
polyA: Homopolymers of adenosine
AAA+: ATPases associated with various cellular activities
ORC: Origin Recognition Complex
MCM: Minichromosome maintenance proteins
ORB: Origin recognition box
*Ap*Orc1: *Aeropyrum pernix* Orc1-1 protein
EMSA: Electrophoretic mobility shift assay
SPR: Surface plasmon resonance
BLI: Bio-layer interferometry
MST: Microscale thermophoresis
