# Supplementary material for: Archaeal Orc1 protein interacts with T-rich single-stranded DNA
Source: BMC Res Notes. 2021 Jul 19;14:275. doi: 10.1186/s13104-021-05690-w (PMC8287685; doi:10.1186/s13104-021-05690-w)
Supplement: Supplementary file 1 — Additional file 1: Figure S1. Sequence alignment of archaeal origin regions. The sequences of origin regions of A. pernix (NC_000854), Pyrococcus abyssi (NC_000868), Pyrococcus furiosus (NC_003413) and Sulfolobus solfataricus (NC_003106) were chosen based on the work of Robinson and co-authors [26]. The sequence alignment was done with the use of Clustal Omega tool [27]. The sequences of the Orc proteins’ binding sites (ORB sites) are bolded. Figure S2. Purification of ApOrc1 protein. The ApOrc1 protein was purified as a His6-tagged variant from the E. coli Rosetta LysS strain. After overproduction of ApOrc1 protein and cells lysis, a fraction of soluble proteins was applied onto a Ni–NTA resin. After the washing step, the protein bound to the resin was eluted with buffer containing 250 mM EDTA (see Materials and Methods). 5 µl of collected fractions were mixed with Laemmli buffer and separated in 12.5% polyacrylamide gel stained with Coomassie Blue dye. M—PageRuler™ Prestained Protein Ladder (Thermo Scientific) (lane 1); S—fraction of soluble proteins (lane 2); FT- fraction of proteins that did not bind to the resin (line 3); E- fractions collected during elution of proteins from the resin (lanes 4–8). Figure S3. Binding of Ori1 region by ApOrc1 protein. Analysis of ApOrc1 protein binding to dsDNA with EMSA technique. Binding to dsDNA fragments containing sequence of Ori1 region (A) or non-specific sequence (fragment of pUC18 plasmid) (B) was tested. (A, B) Fluorescently labeled dsDNA fragment (1 pmol) was incubated with increasing concentration of ApOrc1 protein (100, 200, 300 nM) and then separated in 5% PAGE in TBE buffer. Black arrows indicate the nucleoprotein complexes (A). Black asterisk indicates fuzzy band of unspecific nucleoprotein complex (B). Figure S4. Binding of E. coli SSB protein to ssDNA fragments containing sequence of top and bottom strand of the right part of the AT-rich region of Ori1. Binding was analyzed with SPR technique. Biotinylated ssDN [file 13104_2021_5690_MOESM1_ESM.pdf]

Additional File 1

Archaeal Orc1 protein interacts with T-rich single-stranded DNA

Katarzyna Wegrzyn[1*], Igor Konieczny[1]

[1]Intercollegiate Faculty of Biotechnology of University of Gdansk and Medical University of Gdansk, University of Gdansk, Abrahama 58, 80-307 Gdansk, Poland

*correspondence: katarzyna.wegrzyn@ ug.edu.pl

Materials and Methods

Cloning of gene for *Ap*Orc1

Gene for *Ap*Orc1 protein was cloned into *Nde*I and *Eco*RI restriction sites of the pET15b cloning vector (Novagen) using In-Fusion® HD Cloning Plus kit (Takara Bio) according to the manufacturer's procedure. For amplification of the *Aporc1* gene the primers *Aporc1* top and *Aporc1* bottom (Table S1) were used.

*Ap*Orc1 protein purification

The *Ap*Orc1 protein was purified as a His_6-tagged variant from the *E. coli* Rosetta LysS strain. The cells were grown in LB at 32°C to an OD_{600} of 0.6 and then the gene expression was induced using 0.5 mM IPTG at 25°C for 20 h. Next, cells were harvested by centrifugation and resuspended in buffer containing 50 mM KPi pH 8, 600 mM NaCl, 10 mM imidazole, 10% glycerol, 0.05% TritonX. The cells were incubated with lysozyme for 20 min, sonicated and the lysates were centrifugated 100 000 x*g* for 20 min. Next, the supernatant was incubated with Ni-NTA resin for 1 h at 4°C. Unbound proteins were washed with buffer containing 40 mM imidazole and eluted with 250 mM EDTA. The protein solution was dialyzed to a buffer containing 50 mM KPi pH 8, 200 mM NaCl, 2 mM β-mercaptoethanol, 10% glycerol.

Preparation of DNA probes and Electrophoretic Mobility Shift Assay (EMSA)

The dsDNA fragment containing Ori1 sequence was amplified using Ori1 top and Ori1 bottom primers (Supplementary Table S1) and CloneAmp™HiFiPCR Premix (Takara). The unspecific dsDNA fragments were prepared by hybridization of two oligonucleotides containing sequence of two strands of a fragment of the pUC18 plasmid. After purification of PCR product from an agarose gel and hybridization of the oligonucleotides these dsDNA fragments were labeled with Alexa555-dCTP (Thermo Scientific) and Terminal Deoxynucleotidyl Transferase

(Promega) according to manufacturer's procedure. The fluorescently labeled with Cy3 dye polyT($_{70}$) ssDNA was commercially synthesized (Metabion).

Reaction mixtures containing 1 pmol of the fluorescent DNA probe and increasing amount of ApOrc1 protein (100, 200, 300 nM), in a buffer containing 40 mM HEPES-KOH pH 8, 25 mM Tris-HCl pH 7.6, 100 mM NaCl, 4% (w/v) sucrose, 4 mM dithiothreitol, 80 µg/mL BSA, 10 mM $Mg(OAc)_2$ and 2 mM ATP were incubated for 20 min at 32ºC or 70ºC. After incubation 2.5% (v/v) Ficoll 4000 was added and reactions were loaded onto a 5% polyacrylamide gel. Gels were prepared in Tris-borate/EDTA buffer and, after electrophoresis, scanned with a ChemiDoc (BioRad).

Bio-layer interferometry (BLI)

Standard Bio-layer interferometry (BLI) analyses using BLItz device (ForteBio) were performed at room temperature with 1500 rpm mixing as described in the manufacturer's manual. DNA binding by *Ap*Orc1 proteins was studied using a 5′-biotinylated ssDNA fragments, immobilized on a streptavidin coated SA biosensors (ForteBio). All biotinylated oligonucleotides were synthesized commercially (Metabion, Germany) (Supplementary Table S1). Before each analysis the biosensor was hydrated in HBS-EP buffer (150 mM NaCl, 10 mM HEPES pH 7.4, 3mM EDTA, 0.05% Surfactant P20) (Cytiva) for 10 min, then the baseline was detected for 2 min and 80 nM ssDNA solution was used to immobilize ssDNA for 2 min (Figure 2A, step 1). Next, the unbound ssDNA was washed out for 30 sec in HBS-EP and 30 sec in EDBS buffer (25 mM Tris-HCl [pH 8], 4% [w/v] sucrose, 4 mM DTT, 80 µg/mL BSA, 10 mM $Mg(OAc)_2$ and 2 mM ATP) (Figure 1B, step 2), followed by incubation of the biosensor with 250 nM of the *Ap*Orc1 in EDBS containing buffer for 2 min (Figure 1B, step 3) and EDBS buffer only for sequential 2 min (Figure 1B, step 4). In control reaction, incubation of biosensor with ssDNA solution was

replaced by incubation with only HBS-EP buffer. The obtained data were exported and presented as sensograms showing association (Figure 2A, step 3) and dissociation (Figure 2A, step 4) of the *Ap*Orc1 protein.

Surface plasmon resonance (SPR)

Standard surface plasmon resonance (SPR) analyses using Biacore T200 (Cytiva) were performed as described in the manufacturer's manual. DNA binding by the *Ap*Orc1 proteins was studied using a 5′-biotinylated DNA fragments, immobilized on a streptavidin matrix-coated Sensor Chip SA (Cytiva). All biotinylated oligonucleotides were commercially synthesized (Metabion, Germany) (Supplementary Table S1). The dsDNA fragment containing *Ori1* sequence was amplified using Ori1 top and Ori1 bottom primers (Supplementary Table S1) and CloneAmp™HiFiPCR Premix (Takara), and after purification from an agarose gel, biotinylated with Biotin-14-dCTP (Thermo Scientific) and Terminal Deoxynucleotidyl Transferase (Promega) according to the manufacturer's procedure. Labeling of oligonucleotides with Biotin-14-dCTP (Thermo Scientific) and Terminal Deoxynucleotidyl Transferase (Promega) was also performed for the polyT$_{(60)}$ and polyA$_{(60)}$, according to the manufacturer's procedure. DNA fragments were immobilized on the sensor surface to yield a final value of ~40 RU for a dsDNA fragment containing a sequence of Ori1, ~95 RU for ssDNA containing R and L sequence as well as polyT$_{(60)}$ and polyA$_{(60)}$ and to ~50 RU for ssDNA fragments containing a R1 and R2 sequences. Experiments were run at 25°C and the running buffer was HBS-EP (150 mM NaCl, 10 mM HEPES pH 7.4, 3 mM EDTA, 0.05% Surfactant P20) supplemented with 10 mM $MgCl_2$ and 2 mM ATP. The concentration of ATP was chosen based on a previous publications concerning replication initiator binding with ssDNA [1] and titration analysis, in which increasing concentration of ATP (from 0.03 mM to 4 mM) was used (Figure

S10). Where indicated, instead of ATP the 2mM AMP-PMP or 2mM ADP was added or nucleotide was omitted from the mixture. In experiments the buffer flow rate was set to 15 µl/min. The obtained data were analyzed using Biacore T200 Evaluation Software (Cytiva, USA). The results are presented as sensorgrams obtained after subtraction of the background response signal from a reference flow cell and from a control experiment with buffer injection.

Microscale thermophoresis (MST)

Microscale thermophoresis was performed using the Monolith NT.115 instrument (NanoTemper Technologies GmbH) [25]. Binding between fluorecently labeled ssDNA fragments: polyT$_{(70)}$ and polyA$_{(70)}$ or R top and bottom strands of the AT-rich region of *A. pernix* Ori1, and the *Ap*Orc1 protein was measured. The labeled polyT$_{(70)}$ and polyA$_{(70)}$ were commercially synthesized (Metabion) and labeled R top and bottom strands ssDNA fragments were prepared with Alexa555-dCTP (Thermo Scientific) and Terminal Deoxynucleotidyl Transferase (Promega) according to manufacturer's procedure. A 16-step dilution series of *Ap*Orc1 protein (12 µM) was prepared in EDBS buffer (25 mM Tris-HCl [pH 8], 4% [w/v] sucrose, 4 mM DTT, and 80 µg/mL BSA) supplemented with 10 mM $MgCl_2$ and 2 mM ATP. Next, 10 µL of 80nM labeled ssDNA diluted in EDBS buffer was added to 10 µL of protein solution (1:1 dilution series). The samples were incubated at 32°C for 20 min and centrifugated before being transferred to Standard Monolith NT™ Capillaries. The capillaries were scanned and the fluorescence was measured at 25°C using the MST instrument (medium MST power). For each ssDNA fragment, at least three independent experiments were performed. All data were analyzed using MO Affinity Analysis software (NanoTemper Technologies GmbH) and are presented as dependence of change in the measured fluorescence from protein

concentration. . The increase in a change in a measured fluorescence, when more protein is in the mixture, indicates complex formation.

Supplementary References

6. Wegrzyn, K., et al., *Sequence-specific interactions of Rep proteins with ssDNA in the AT-rich region of the plasmid replication origin*. Nucleic Acids Res, 2014. **42**(12): p. 7807-18.

25. Jerabek-Willemsen, M., et al., *MicroScale Thermophoresis: Interaction analysis and beyond.* J Mol Struct, 2014. **1077**: p. 101-113.

26. Robinson, N. P., et al., *Identification of two origins of replication in the single chromosome of the archaeon Sulfolobus solfataricus.* Cell, 2004. **116**(1): p. 25-38.

27. Madeira, F. , et al., *The EMBL-EBI search and sequence analysis tools APIs in 2019.* Nucleic Acids Res, 2019. **47**(W1):W636-W641.

Table S1. Oligonucleotides used in this study.

Oligonucleotide	Sequence
L top	5'(biotin) GAAGCGTATACTCCTCCCGAATAGTGCTCTTGAAGAATGCCAATATAAT GATACAAAAACCCAGTTTTAAA 3'
L bottom	5'(biotin) TTTAAAACTGGGTTTTTGTATCATTATATTGGCATTCTTCAAGAGCACTA TTCGGGAGGAGTATACGCTTC3'
R top	5'(biotin) CAAATCAAGCTAGAAACAAACAAAGACACA CTATAATCCAGAACTGCA CAGCCTGA3'
R bottom	5'(biotin) TCAGGCTGTGCAGTTCTGGATTATAGTGTGTCTTTGTTTGTTTCTAGCTTG ATTTG3'
L1 top	5'(biotin) GAAGCGTATACTCCTCCCGAATAGTGCTCTTGAAGA 3'
L1 bottom	5'(biotin) TCTTCAAGAGCACTATTCGGGAGGAGTATACGCTTC 3'
L2 top	5'(biotin) ATGCCAATATAATGATACAAAAACCCAGTTTTAAA 3'
L2 bottom	5'(biotin) TTTAAAACTGGGTTTTTGTATCATTATATTGGCAT 3'
R1 top	5'(biotin) CAAATCAAGCTAGAAACAAACAAAGACACA 3'
R1 bottom	5'(biotin) TGTGTCTTTGTTTGTTTCTAGCTTGATTTG 3'
R2 top	5'(biotin) CTATAATCCAGAACTGCACAGCCTGA 3'
R2 bottom	5'(biotin) TCAGGCTGTGCAGTTCTGGATTATAG 3'
$polyT_{(70)}$	5’ Cy3-TTTTTTTTTT TTTTTTTTTT TTTTTTTTTT TTTTTTTTTT TTTTTTTTTT TTTT TTTTTT TTTTTTTTTT 3’
$polyA_{(70)}$	5’ Cy3-AAAAAAAAAA AAAAAAAAAA AAAAAAAAAA AAAAAAAAAA AAAAAA AAAA AAAAAAAAAA AAAAAAAAAA 3’
$polyT_{(60)}$	5’ TTTTTTTTTT TTTTTTTTTT TTTTTTTTTT TTTTTTTTTT TTTTTTTTTT TTTTTTTT TT 3’
$polyA_{(60)}$	5’ AAAAAAAAAA AAAAAAAAAA AAAAAAAAAA AAAAAAAAAA AAAAAAAAA A AAAAAAAAAA 3’
Ori1 top	5' TTAGGATGCTCCACAGGAAACGGAG 3'
Ori1 bottom	5' CCCCCCGGTATACGACCCC 3'
ssDNA uspec.	5'(biotin) GCA GCC CTG GTT AAA AAC AAG GTT TAT AAA TAT TGG TTT AAA AGC AGG TTA AAA GAC AGG TTA GCG GTG G 3’
pUC18 fragment top	5’AGCTCACAATTCCACACAACATACGAGCCGGAAGCATAAAGTGTAAAGCCTGGG GTGCCTAATGAGTGAGCTAACTCACATTAATTGCGTTGCGCTCACTGCCCGCTTTCC AGTCGGGAAACCTGTCGT 3’

pUC18 fragment bottom	5'ACGACAGGTTTCCCGACTGGAAAGCGGGCAGTGAGCGCAACGCAATTAATGTGA GTTAGCTCACTCATTAGGCACCCCAGGCTTTACACTTTATGCTTCCGGCTCGTATGT TGTGTGGAATTGTGAGCT 3'
Aporc1 top	5' GCCGCGCGGCAGCCATATG GAGGAGGTTTTCACGGC 3'
Aporc1 bottom	5' TCGTCTTCAAGAATTC TTATAACAGCCTGGCTACGAAG 3'

Figure S1

```
A. pernix       ------------------------------------------------------------ 0
P. abyssi       ------------------CATTTTTACACA------------------------------ 12
P. furiosus     TACTTAATATGGGAGAGTGTTTGTCACCAGAAAAAATTTATTTTTTCTGGTGTTTGCCGC 60
S. solfataricus ------------------------------------------------------------ 0

A. pernix       ------------------------------------------------------------ 0
P. abyssi       --------------AATAAGTGCATTAGGTTAACTTAATTTGTTTAATATTTTGCCAAAG 58
P. furiosus     CCGGCTCCAGTGGAAATGAAACTCTGGGGGACTTCACTTCTGTTTAATAATTGGAAAATG 120
S. solfataricus -------------------------------TAAGACGGATGTTATTTAACTGGGGAGTA 29

A. pernix       ---------TTAGGATGCTCCACAGGAAACGGAGGGG----------------------- 28
P. abyssi       TTAAACAGAA------------------------------------------GTGAAGTC 76
P. furiosus     TTAAACAAATTAGATTAATCTAATGAACTAAAAGGTGCAATAATGAACAAATTTTAATGA 180
S. solfataricus AAAAA------------------------------------------------------- 34

A. pernix       ----------TCCTTATAAACCCCCGGCAACCACGCCACGCCGCACCCCGCAGGAAAGCC 78
P. abyssi       CCCCAG-----GGTTTCATTTCCACTGGAACCGGGTTGCGACACCGAGAATATTCTATTC 131
P. furiosus     CCATAAATGTTCATTTTGTCTCCACAGGAAATCTGAAGTTCCTGTAGAAATATGTAAACA 240
S. solfataricus ----------CCCCTTTGTTTCCACTGGAGAACTTAAGGTAAATATAAATTAAGTTTACC 84
                              *      ** * * *                      *

A. pernix       CCACACCCC-------------CCAGGTTCCTCTGGAGCATCAGAAGCGTATACTCCTCC 125
P. abyssi       TTTTTTCTGACAAGACACTCTCCCATATTAGTTGGAACTCCTATTTAAATATTTATCTAT 191
P. furiosus     TCAAATGTC----------CAAAGATGTTCATT----CGCTTATCTC----ATTTCCTGT 282
S. solfataricus AGAGACCTA----------C---------------------------------------- 94

A. pernix       CGAATAGTGCTCTTGAAGAATGCCAA-TATAATGATACAAAAACCCAGTTTTAAACAAAT 184
P. abyssi       GAACAGTTTTATAAGTAAATTGAGAAGCAGAGAAGCAAATGTTCACATATTTATGCACAA 251
P. furiosus     GGAAGTTT-TGGGCTGGGTGTGGAACTTCCACTGGAAAATATTGACATGTTTTTGTCAAG 341
S. solfataricus -------------------------------------------CCCATTGTTTCGTTTGG 111
                                                             **   **

A. pernix       CAAGCTAGA--AACAAACAA-----AGACACACTATAATCC------------------- 218
P. abyssi       AATAGTACATCAACTGAAAA---------AATGTTCATTTG------------------- 283
P. furiosus     CAAAGGATAAAAACTCAACAGTTAAAGGAATAGTTGCCTTTCCAATGTTTCCTCTATTTC 401
S. solfataricus AAAATTATTAATATGATATAAGCCTA-GAATTGTCTTCTTATCAA----TTCCTTTTGTA 166
                 *    *     *      *         *   *    *

A. pernix       ------AGA----------------------ACT-------------GCACAGCCTGAGA 237
P. abyssi       ------CAAAAGTAAAAATTTTTGGCTTTTAATT-----------------TTACGTTTG 320
P. furiosus     CAATGGAGATACTATATATCTATGGCATTTATCATAACTAAATTATTACATTTAGTTATA 461
S. solfataricus TAATGTTTATCTTATTTATCTC---------AATTAGCTAGAT--------TAAGTAATT 209
                        *

A. pernix       GGATCCAGCGGAAAACAGG----GGGTGTGGGGG---------------------CTTAG 272
P. abyssi       TAAACCAACGAAGAATTGC----ATGCCTTTCTCAACCCAGTTCCACTG---GAAATGAA 373
P. furiosus     TGGATGGGGGAAAAATTAACAACATGTGTTATGTTTC-CTCTGGAAAATTGATCTATAAT 520
S. solfataricus TCCAGAGGAAATAGAT---------GGGTCCCACTTCGCTTTTAAATGGTATTTTGTGGA 260
                              *          *  *                           *

A. pernix       TGTGTCAGCTCGCCGTCTCCACAGGAAACGG----------------AGGGGTCGTATAC 316
P. abyssi       ACTCTGGGGGGTTCTTTA------GATGGCTATCTGAATGCTAGCATATAAAT------- 420
P. furiosus     AATCTAGGAGCACAATTTCCAATGGAGGGTCATCAATGAACGAAGGTGAACATCAAATAA 580
S. solfataricus CTTGTAGAATTTTCATTAAGAGTTAAGAGGCATTATAAACCTAAATTGGACATCTAATCA 320
                  * *          *         *                          *

A. pernix       CGGGGGG-------------------------------------------   323
P. abyssi       ----------------CGAAAATATACAAGAAAATGTCAAAAAAAT----   450
P. furiosus     AGCTTGACGAGCTATTCGAAAAGTTGCTCCGAGCTAGGAAGATATTCAAA   630
S. solfataricus ATAATTGAAAGTTAATAGTAGATCAG------------TAAA--------   350
```

Figure S1. Sequence alignment of archaeal origin regions. The sequences of origin regions of *A. pernix* (NC_000854), *Pyrococcus abyssi* (NC_000868), *Pyrococcus furiosus* (NC_003413) and *Sulfolobus solfataricus* (NC_003106) were chosen based on the work of Robinson and co-authors [26]. The sequence alignment was done with the use of Clustal Omega tool [27]. The sequences of the Orc proteins' binding sites (ORB sites) are bolded.

Figure S2

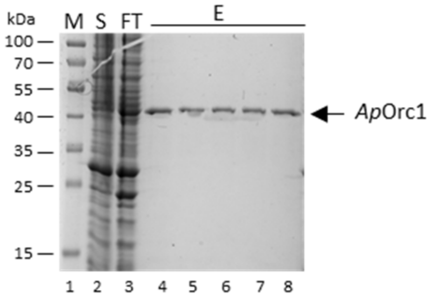

Figure S2. Purification of *Ap*Orc1 protein. The *Ap*Orc1 protein was purified as a His6-tagged variant from the *E. coli* Rosetta LysS strain. After overproduction of *Ap*Orc1 protein and cells lysis, a fraction of soluble proteins was applied onto a Ni-NTA resin. After the washing step, the protein bound to the resin was eluted with buffer containing 250 mM EDTA (see Materials and Methods). 5µl of collected fractions were mixed with Laemmli buffer and separated in 12.5% polyacrylamide gel stained with Coomassie Blue dye. M - PageRuler™ Prestained Protein Ladder (Thermo Scientific) (lane 1); S - fraction of soluble proteins (lane 2); FT- fraction of proteins that did not bind to the resin (line 3); E- fractions collected during elution of proteins from the resin (lanes 4-8)

Figure S3

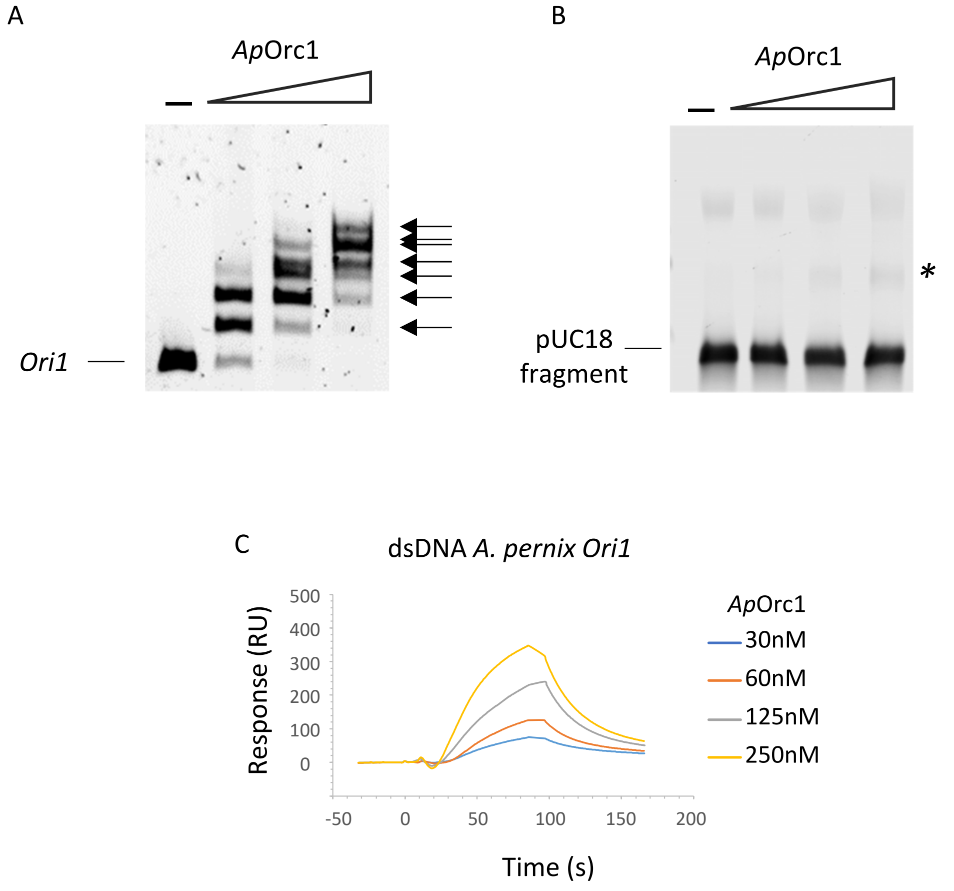

Figure S3. Binding of *Ori1* region by *Ap*Orc1 protein. Analysis of *Ap*Orc1 protein binding to dsDNA with EMSA technique. Binding to dsDNA fragments containing sequence of Ori1 region (A) or non-specific sequence (fragment of pUC18 plasmid) (B) was tested. Analysis of *Ap*Orc1 protein binding to dsDNA containing sequence of Ori1 region with SPR techniques (C). (A, B) Fluorescently labeled dsDNA fragment (1pmol) was incubated with increasing concentration of *Ap*Orc1 protein (100, 200, 300nM) and then separated in 5% PAGE in TBE buffer. Black arrows indicate the nucleoprotein complexes (A). Black asterisk indicates fuzzy band of unspecific nucleoprotein complex (B) (C) Biotinylated dsDNA fragments were immobilized on a surface of sensor chip SA. Injections containing the indicated concentrations of protein in HBS-EP buffer were performed. HBS-EP was also used as a running buffer.

Figure S4

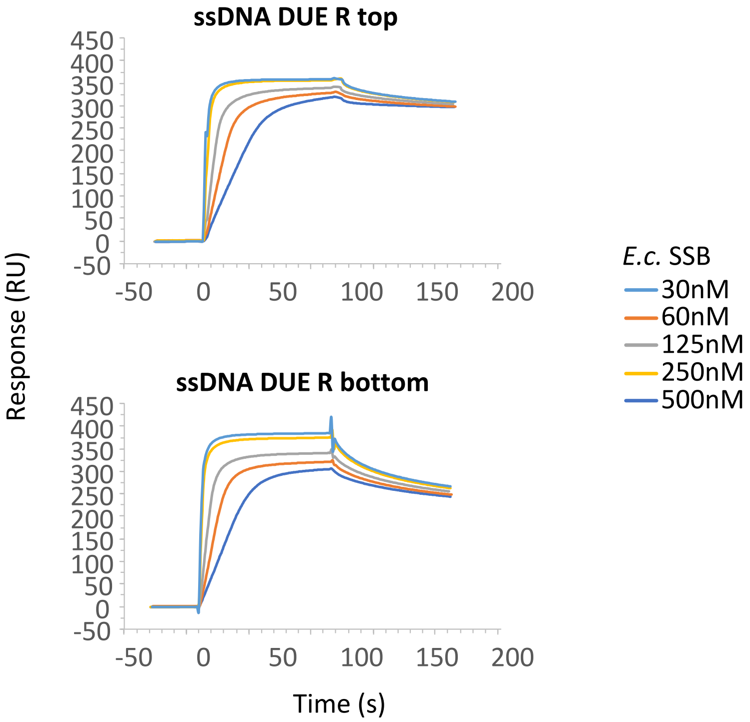

Figure S4. Binding of *E. coli* SSB protein to ssDNA fragments containing sequence of top and bottom strand of the right part of the AT-rich region of *Ori1*. Binding was analyzed with SPR technique. Biotinylated ssDNA fragments (Table S1) were immobilized on a surface of sensor chip SA. Injections containing the indicated concentrations of SSB protein in HBS-EP buffer were performed. HBS-EP was also used as a running buffer.

Supplementary Figure S5

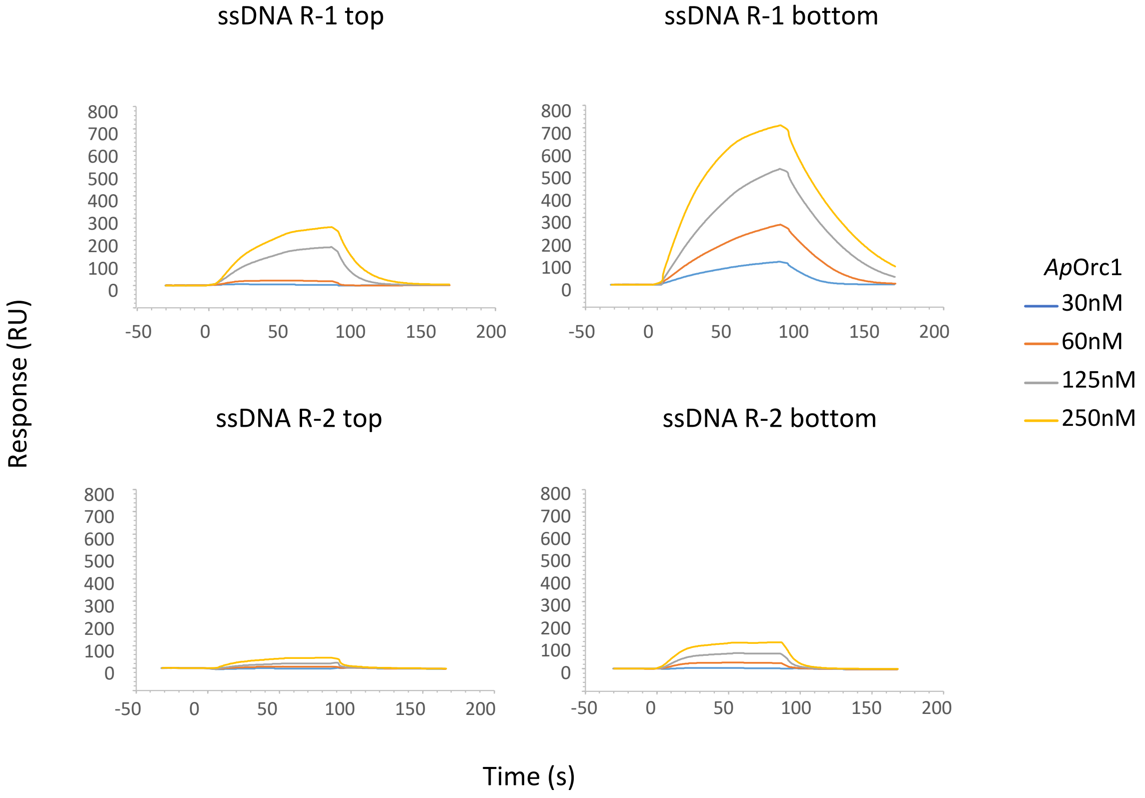

Figure S5. Binding of *Ap*Orc1 protein to ssDNA fragments containing sequence of top and bottom strand of right part of the AT-rich region of *Ori1*. Binding of the *Ap*Orc1 protein to indicated ssDNA fragments containing sequence of right part of the AT-rich region of *Ori1* was analyzed with SPR technique. Biotinylated ssDNA fragments (Table S1) were immobilized on a surface of sensor chip SA. Injections containing the indicated concentrations of protein in HBS-EP buffer were performed. HBS-EP was also used as a running buffer.

Figure S6

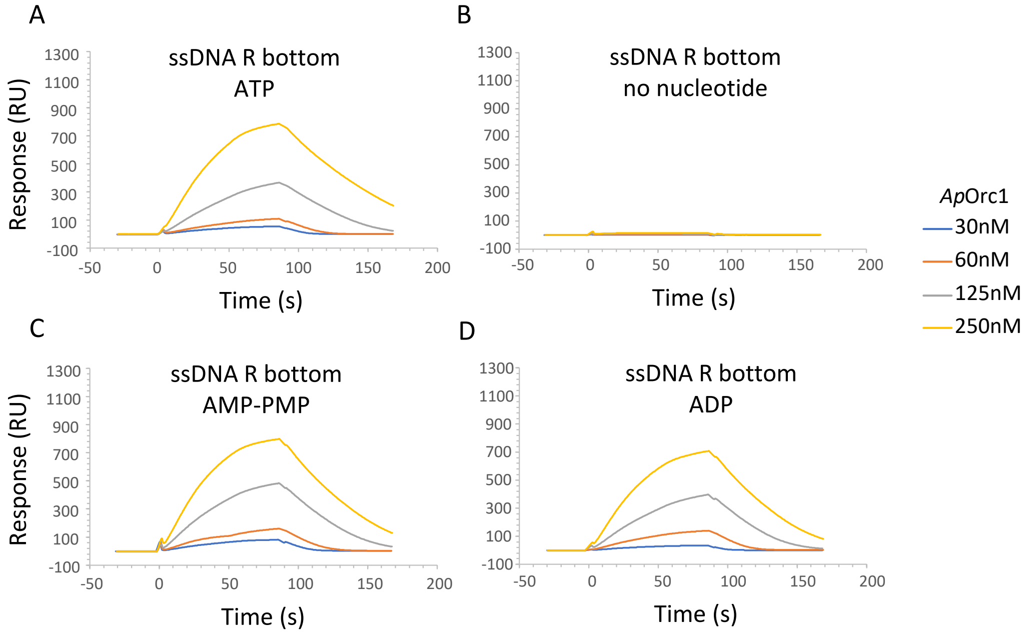

Figure S6. Influence of nucleotides on *Ap*Orc1 protein binding to ssDNA. Binding of the *Ap*Orc1 protein to ssDNA fragments (Table S1) containing right bottom strands' sequence of the Ori1 AT-rich region was analyzed with SPR technique. Biotinylated ssDNA fragments were immobilized on a surface of SA sensor chip. Injections containing the indicated concentrations of protein in HBS-EP buffer with (A) or without (B) ATP, with AMP-PMP (non-hydrolyzable analogue of ATP) (C) and ADP (D) were performed. HBS-EP was used as a running buffer.

Figure S7

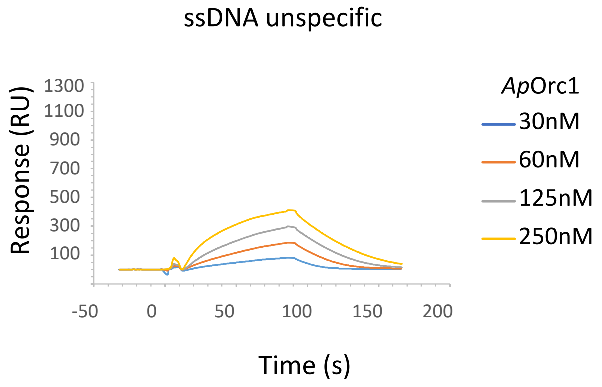

Figure S7. Binding of *Ap*Orc1 protein to non-specific ssDNA fragment. Binding of *Ap*Orc1 protein to ssDNA fragments (Table S1) containing sequence of AT-rich region of plasmid RK2 was analyzed with SPR technique. Biotinylated ssDNA fragments were immobilized on a surface of sensor chip SA. Injections containing the indicated concentrations of protein in HBS-EP buffer were performed. HBS-EP was also used as a running buffer.

Figure S8

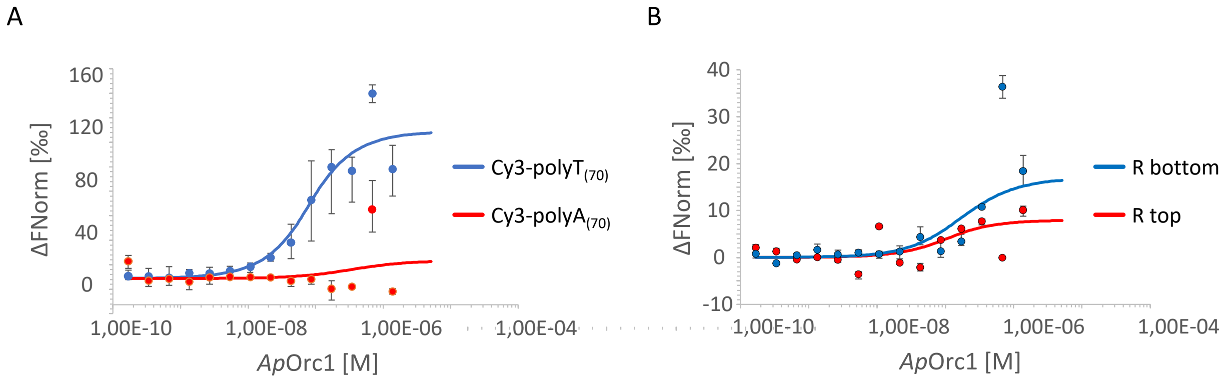

Figure S8. MST analysis of *Ap*Orc1 binding to ssDNA fragments. Binding was analyzed with MST technique. Fluorescently labeled ssDNA fragments containing sequence of polyT$_{(70)}$ (A, blue) and poly A$_{(70)}$ (A, red) or sequence of R bottom (B, blue) and R top strands (B, red) of AT-rich region of *Ori1* (Table S1) were mixed with increasing concentration of ApOrc1 protein in EDBS buffer. Mixtures were transferred to Standard Monolith NT™ Capillaries and fluorescence was measured with Monolith NT.115. For each ssDNA fragment, at least three independent experiments were performed. The obtained data were analyzed with MO.Affinity Analysis software and presented as dependence of change in the fluorescence from protein concentration.

Supplementary Figure S9

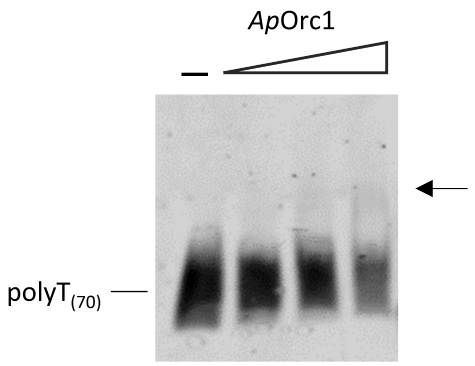

Figure S9. Binding of polyT ssDNA by ApOrc1 protein. Binding of *Ap*Orc1 protein to homopolymer of thymidine (polyT$_{(70)}$) was analyzed with EMSA technique. Fluorescently labeled ssDNA fragment (1 pmol) was incubated with increasing concentration of *Ap*Orc1 protein (100, 200, 300 nM) and then separated in 5% PAGE in TBE buffer. Black arrow indicates the nucleoprotein complex.

Figure S10

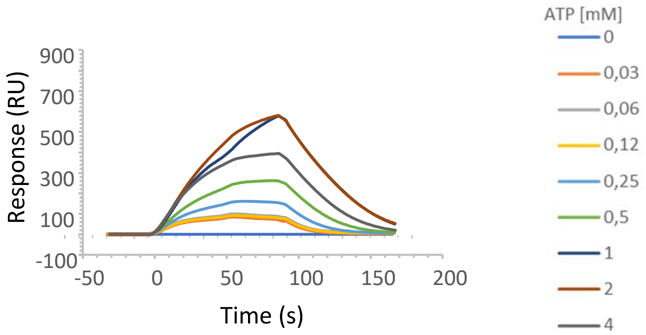

Figure S10. Dependence of *Ap*Orc1 binding to R bottom ssDNA fragments from ATP concentration. Binding was analyzed with SPR technique. Biotinylated ssDNA fragments containing sequence of R bottom strand of AT-rich region of Ori1 (Table S1) were immobilized on a surface of sensor chip SA. Injections containing the constant concentrations of ApOrc1 (125nM) protein in HBS-EP buffer supplemented with increasing concentration of ATP were performed. HBS-EP was also used as a running buffer.
